# Knowledge status of skin tear prevention and its demographic and occupational influencing factors: A National cross‐sectional survey among nurses

**DOI:** 10.1111/jan.16353

**Published:** 2024-07-23

**Authors:** Qingli Jiang, Huilin He, Ke Jing, Miyan Wang, Xiaochun He, Rong Hu, Yuwei Yang, Fang He

**Affiliations:** ^1^ Mianyang Central Hospital, Affiliated with the School of Medicine University of Electronic Science and Technology of China Mianyang China; ^2^ NHC Key Laboratory of Nuclear Technology Medical Transformation (Mianyang Central Hospital) Mianyang China

**Keywords:** knowledge, nurses, self‐directed learning competence, skin tears

## Abstract

**Aim:**

A skin tear (ST) is a common skin injury that is often misdiagnosed or overlooked. This study examined the current state of nurses' ST knowledge and its influencing factors.

**Design:**

A national cross‐sectional survey combined with a quantitative analysis was used to provide evidence of poor ST knowledge among nurses and its influencing factors.

**Methods:**

An electronic questionnaire survey was conducted among 1293 nurses from 32 hospitals in 18 provinces across China, including a General Information Questionnaire, ST Knowledge Assessment Instrument (OASES) and a Self‐directed Learning Competence Scale for Nurses (SLCS‐N).

**Results:**

The mean OASES score was 9.51 ± 3.15, with a score rate of 47.55%. Pearson's correlation analysis showed positive correlations, ranging from none to strong, between every dimension in the OASES and from strong to extremely strong between every dimension in the SLCS‐N. Multivariate analysis revealed multiple independent factors influencing ST knowledge, such as hospital tier, specialized nurses in wound/ostomy/incontinence care, participation in training for wound/ostomy/incontinence management, willingness to undergo ST training, self‐assessed grade in ST care and the degree of emphasis of managers.

**Conclusion:**

ST knowledge status was generally poor among nurses nationwide. Managers should establish a comprehensive and specialized curriculum‐based system, develop evidence‐based standardized nursing processes, and provide tailored training programs to address nurses' unique characteristics and individualized needs, thereby enhancing their proficiency in ST‐related knowledge and skills.

**Impact:**

This study is the first to identify a poor level of ST knowledge among nurses nationwide, particularly in the four dimensions of risk assessment: prevention, treatment, classification, and observation. Based on the findings regarding demographic factors and ST experiences, an integrated management system and educational program should be implemented to improve nurses' awareness and knowledge in this field.

**Patient or Public Contribution:**

No patient or public contributions.

## 1 INTRODUCTION

A skin tear (ST) is a traumatic wound caused by mechanical forces, such as blunt trauma, falls, improper equipment operation, equipment damage, or removal of medical adhesive dressings (Holloway et al., 2023). They are a prevalent type of skin injury in hospitalized patients (Völzer et al., 2023), with incidence rates ranging from 3.3% to 19.8% in general hospitals, 3.0%–26% in long‐term care centers, and 5.5% to 19.5% in community hospitals (Bermark et al., 2018; Miles et al., 2022; Minematsu et al., 2021; Serra et al., 2018; Souza et al., 2021; Van Tiggelen et al., 2020). STs primarily occur in older individuals, newborns, critically ill patients and those with chronic illnesses (LeBlanc et al., 2018; LeBlanc et al., 2023), and predominantly affect the upper and lower limbs (approximately 80%) (Bandeira et al., 2022; Chang et al., 2016). STs as hospital‐acquired adverse events have been widely reported, resulting in patient discomfort, prolonged hospitalization, increased healthcare expenses, diminished quality of life, reduced patient satisfaction and compromised quality of care (Hardie, 2020). Particularly for patients with lower limb injuries and/or multiple comorbidities, STs are prone to develop into challenging chronic wounds that can result in wound infection or even systemic infection, thereby posing a significant risk to life. This issue has become a crucial concern that affects patient safety in the hospital setting (Hardie & Wick, 2020; Stephen‐Haynes, 2020).

However, the recognition of STs occurred relatively late. ST was first defined in 1993 by Payne et al (Payne & Martin, 1993), subsequently, the International Skin Tear Advisory Panel (ISTAP) was established in 2011 to address this issue (LeBlanc & Baranoski, 2011). The ISTAP released the first best practices for the prevention and management of STs in 2018 (LeBlanc et al., 2023), and China did not have the first edition of expert consensus until 2023 (Chinese Geriatrics Society Burn and Trauma Branch, 2023). These best practices and expert consensus have identified a variety of risk factors for STs, including intrinsic factors such as skin aging and malnutrition, as well as extrinsic factors such as falls and adhesive use (Cilluffo et al., 2023; Stephen‐Haynes, 2023). Early risk identification, timely prevention, and treatment are highlighted as key strategies. However, in clinical settings, nurses often lack attention and recognition of STs, leading to misjudgment and under‐reporting. In addition, insufficient knowledge and skills in prevention and care result in treatment delays, ultimately creating a significant gap between current clinical practices and best practices (Chinese Geriatrics Society Burn and Trauma Branch, 2023; LeBlanc et al., 2014).

Due to the late recognition of STs, current studies have primarily focused on investigating prevalence (Bandeira et al., 2022; Bermark et al., 2018; Koyano et al., 2016; LeBlanc et al., 2014; Miles et al., 2022; Yüceler Kaçmaz et al., 2022), analysing risk factors (Koyano et al., 2016; Serra et al., 2018; Soh et al., 2019), constructing risk prediction models (Bermark et al., 2018; Rayner, Carville, Leslie, & Dhaliwal, 2019; Soh et al., 2019; Van Tiggelen et al., 2019), and validating classification systems (Bassola et al., 2019; Van Tiggelen et al., 2017). Few studies have examined the comprehensive knowledge of nurses in preventing and managing STs (Formosa et al., 2022; Kaçmaz et al., 2023; Scheele et al., 2020). Christa et al. developed a questionnaire for their investigation; however, the reliability of their conclusions requires further confirmation (Scheele et al., 2020). In 2020, ISTAP developed the OASES (ST knowledge assessment instrument) (Van Tiggelen et al., 2021). The validation across 37 countries demonstrated that the scale had appropriate difficulty overall, good discrimination ability, and reliability and validity; thus, it can be used to evaluate the knowledge level of nurses on STs (Feng, Hu, Li, Ying, et al., 2023; Hu et al., 2022; Luo et al., 2023). A survey of 346 nurses revealed a low level of knowledge regarding STs in terms of aetiology, classification, observation, risk assessment, prevention, and treatment (Kaçmaz et al., 2023). A survey conducted among nurses in Class III Grade A hospitals in China indicated that only 39.8% achieved qualified scores of 60% for ST knowledge (Stephen‐Haynes, 2020). However, this survey did not consider the knowledge level of nurses in hospitals at other levels. The aim of the current study was to employ the OASES to conduct a national survey of ST knowledge of nurses and analyse its influencing factors, thereby providing a foundation for the subsequent development of relevant nursing standards and training programs.

## 2 METHODS

### 2.1 Ethical review

This study was approved by the Ethics Committee of Mianyang Central Hospital (Approval number S202303119‐01; December 27, 2023) and conducted in compliance with the principles of the Declaration of Helsinki. Nurses were unable to participate in the survey until they provided informed consent.

### 2.2 Study design and implementation

This nationwide cross‐sectional survey involved 32 hospitals in all hospital tiers (Class I to III, Grades A and B) across 18 provinces and cities in North, East, Northeast, South Central, Southwest and Northwest China. The participants were full‐time nurses who held nurse practice qualification certificates and voluntarily participated in the study. The exclusion criteria were nurses engaged in advanced studies, standardized training or internships. The questionnaire included three parts: a general information questionnaire, the OASES, and a Self‐directed Learning Competence Scale for Nurses (SLCS‐N) scale (Hu et al., 2022; Van Tiggelen et al., 2021; Xiao, 2007). The questionnaire comprised 54 items, and at least 648 participants were needed to ensure a ten‐fold increase (Wang et al., 2023) and compensate for a loss of up to 20%.

### 2.3 Data collection

From January to February 2024, after obtaining consent from each representative hospital's nursing department, an electronic questionnaire was distributed via the questionnaire website of *WenJuanXing*. Nurse leaders forwarded an electronic poster containing the QR code for the questionnaire to WeChat workgroup chats, inviting eligible nurses to participate. After scanning the QR code, a signed informed consent was required. After signing the informed consent, they could voluntarily participate in the survey. All participants were required to answer every question and complete the questionnaire within 30 min.

### 2.4 General information questionnaire

A general information questionnaire was developed which included: (1) demographic characteristics, such as sex, age, professional title, number of years worked, educational background, hospital tier and department; (2) ST nursing history, including whether as a wound/ostomy/incontinence specialized nurse, participation in training for wound/ostomy/incontinence care, nursing experience with ST patients, self‐assessed grade in a ST care setting, as a novice, advanced beginner, competent, proficient or expert based on Benner's Theory of Nursing Ability Levels (Benner, 1982), degree of emphasis of managers, as well as previous experiences and willingness to undergo ST training.

### 2.5 ST knowledge assessment instrument(OASES)

The translated OASES was used in this study to examine the level of nurses' knowledge regarding STs (Hu et al., 2022), with permission from the original authors of the OASES tool (Van Tiggelen et al., 2021). The scale encompasses six knowledge domains: aetiology, classification and observation, risk assessment, prevention, treatment, and specific patient groups, comprising 20 items. Each item was presented as a single‐choice question, where one of five options could be chosen, with a correct answer receiving 1 point and an incorrect answer receiving 0 points. The total score ranged from 0 to 20 points, with higher scores indicating a greater understanding of ST knowledge. As the number of items differed in each field, the standard score rate was calculated to ensure comparability among fields. This was determined by dividing the mean score of a field by its total score and multiplying by 100%. The scale demonstrated a difficulty coefficient for all items ranging from 0.24 to 0.94, an intragroup correlation coefficient of 0.83 and a Cohen's Kappa coefficient ranging between 0.21 and 0.74.

### 2.6 SLCS‐N

The SLCS‐N was used to assess the self‐directed learning ability of nurses (Dong & Meng, 2022). This scale includes four dimensions: self‐motivational belief, task analysis, self‐monitor and regulation, and self‐evaluation, comprising 34 items with a 5‐point Likert scale for each item, and total scores ranging from 34 to 170. Higher scores indicated stronger self‐directed learning abilities. The scale had an overall Cronbach's alpha coefficient of .944, with each dimension ranging from 0.701 to 0.887. Additionally, the scale demonstrated an overall split‐half reliability of 0.894, with each dimension ranging from 0.721 to 0.832.

### 2.7 Statistical analysis

SPSS 27.0 was utilized for data processing and analysis, with two‐sided tests and a significance level of *α* = .05. Measurement data were presented as mean ± standard deviation (*SD*), while count data were shown as frequency (percentage). The two‐sample comparisons were performed using independent‐sample t‐tests. The multiple‐sample comparisons were conducted using a one‐sample analysis of variance (ANOVA) if the variance was homogeneous, or Welch's test if there was an inhomogeneity of variance. Pearson's correlation analysis was performed to examine the relationship between ST knowledge scores and self‐directed learning competence scores. When *p* < .05, the absolute values of correlation coefficients (|*r*|) of <.2, .2–.4, .4–.6, .6–.8, and >.8 were considered as no, mild, moderate, strong, and extremely strong correlations, respectively. Finally, multiple linear stepwise regression was used to identify the factors influencing ST knowledge scores.

## 3 RESULTS

### 3.1 General information of nurses (demographic characteristics and ST nursing history)

A total of 1318 eligible nurses completed the online questionnaire. After excluding those with evident errors and invalid responses (where all responses for every item in the questionnaire were identical), 1293 questionnaires were finally retrieved, resulting in an effective recovery rate of 98.1%.

The general information of the participants is detailed in Table S1. Due to the word‐limit, descriptive statistics regarding the demographic characteristics of the nurses are not presented (Table S1). The ST nursing history is as follows: 23.5% (*n* = 204) specialized in wound/ostomy/ incontinence nursing, 57.6% (*n* = 745) had received training related to wound/ stoma/incontinence, 37.1% (*n* = 480) had undergone specialized ST training. A significant majority, 83.1% (*n* = 1075), expressed their willingness to have specialized training in STs; of them, 71.5% (*n* = 924), 56.4% (*n* = 729), and 68.5% (*n* = 886) anticipated independently learning from teaching video materials, online centralized learning, and face‐to‐face intensive training, respectively. A total of 68.4% (*n* = 884) had nursing experience in patients with STs. A total of 90.6% (*n* = 1171) expected a degree of emphasis from managers. The self‐assessed grades of ST nursing ability were as follows: 39.6% (*n* = 512) considered themselves novices, 47.0% (*n* = 608) advanced beginners, 8.0% (*n* = 104) competent, 4.7% (*n* = 61) proficient, and 0.6% (*n* = 8) experts.

### 3.2 ST knowledge status of the nurses

The mean overall score for ST knowledge was 9.51 ± 3.15, with a standardized scoring rate of 47.55%. The standardized scoring rates for the six knowledge domains, ranked from lowest to highest, were: risk assessment, prevention, treatment, classification and observation, aetiology and specific patient groups (Table 1). Table 2 lists the top six items with the lower standardized scoring rates for ST knowledge.

**TABLE 1:** Nurses' scores and standardized scoring rates for ST knowledge in each dimension of OASES (*N* = 1293).

Themes	Total score	M ± SD	Standardized scoring rates (%)
Aetiology	3.00	2.00 ± 0.77	66.67%
Classification and observation	4.00	2.20 ± 1.06	55.00%
Risk assessment	2.00	0.31 ± 0.53	15.50%
Prevention	6.00	2.23 ± 1.31	37.17%
Treatment	4.00	1.93 ± 1.10	48.25%
Specific patient groups	1.00	0.84 ± 0.37	84.00%
OASES score	20.00	9.51 ± 3.15	47.55%

**TABLE 2:** Top six Items with the lower standardized scoring rates for ST knowledge (*N* = 1293).

Dimensions	Items	Standardized scoring rates(%)
Classification and observation	K8 Why are neonates at risk of developing a skin tear?	16%
	K9 Why is the long‐term use of corticosteroids a risk factor for developing a skin tear?	15%
Prevention	K11 Why are skin moisturizers applied to prevent skin tears?	15%
	K13 A humectant (e.g. urea) supports skin hydration. Why?	18%
	K15 What is effective in reducing the risk of developing a skin tear?	30%
Treatment	K19 CASE: Type 2 skin tear (partial flap loss). After cleansing the wound bed, the following observations can be made: T(Thin fibrin layer), I(No signs of local infection), M(Dry wound, no exudate), and E(Fragile surrounding skin). Which treatment do you recommend after cleansing the wound bed?	27%

*Note*: The content of the items is displayed in the form of initial letters of the subscale, item number and specific content.

### 3.3 Comparison of ST knowledge scores of nurses with different demographic characteristics and ST nursing history

With demographic characteristics as independent variables (Table 3), the analysis revealed statistically significant differences in ST knowledge scores among different groups based on sex, age, professional title, number of years worked, educational background, hospital tier, and department (*p* < .05).

**TABLE 3:** Comparative analysis of ST knowledge scores among nurses with different demographic characteristics(*N* = 1293).

Demographic characteristics	*N* (%)	M ± SD	*t/F*	*p*
Sex				
Male	27 (2.10%)	7.67 ± 4.54	−2.145	.041*
Female	1266 (97.90%)	9.55 ± 3.11		
Age(years)				
20–29	375 (29.00%)	8.84 ± 2.97	8.374	<.001*
30–39	664 (51.40%)	9.72 ± 3.17		
40–49	224 (17.30%)	9.95 ± 3.24		
≥50	30 (2.30%)	9.97 ± 3.31		
Professional title				
Nurse	185 (14.30%)	8.76 ± 2.77	12.659	<.001*
Primary nurse	432 (33.40%)	8.97 ± 2.98		
Supervisor nurse	602 (46.60%)	9.99 ± 3.30		
Co‐chief superintendent nurse	59 (4.60%)	10.36 ± 2.84		
Chief superintendent nurse	15 (1.20%)	11.73 ± 2.87		
Number of years worked				
1–5	244 (18.90%)	9.18 ± 2.98	8.305	<.001*
6–10	342 (26.50%)	8.84 ± 3.18		
11–15	346 (26.80%)	9.97 ± 3.00		
16–20	207 (16.00%)	10.05 ± 3.20		
>20	154 (11.90%)	9.77 ± 3.33		
Educational background				
Associate degree	289 (22.40%)	8.83 ± 2.93	11.89	<.001*
Bachelor's degree	981 (75.90%)	9.67 ± 3.20		
Postgraduate degree or above	23 (1.80%)	11.30 ± 2.49		
Hospital tier				
Class III grade A hospital	723 (55.90%)	10.04 ± 3.15	15.847	<.001*
Class III grade B hospital	245 (18.90%)	8.92 ± 3.07		
Class II grade A hospital	177 (13.70%)	9.24 ± 2.98		
Class II grade B hospital	80 (6.20%)	8.79 ± 3.11		
Class I hospital	68 (5.30%)	7.56 ± 2.52		
Department				
Internal medicine	409 (31.60%)	9.64 ± 3.20	5.637	<.001*
Surgical	449 (34.70%)	9.82 ± 2.95		
Gynaecology and obstetrics	26 (2.00%)	8.58 ± 2.66		
Paediatric	37 (2.90%)	8.46 ± 3.42		
ICU	63 (4.90%)	9.02 ± 3.03		
Emergency	28 (2.20%)	9.18 ± 3.23		
Operating theatre	18 (1.40%)	9.33 ± 2.35		
Stoma and wound care	33 (2.60%)	11.89 ± 2.67		
Other comprehensive departments (Rehabilitation department, Infectious disease department, Oncology department, Traditional chinese medicine department)	230 (17.80%)	8.80 ± 3.34		

*Note*: *p<.05.

With ST nursing history as an independent variable (Table 4), the analysis demonstrated statistically significant differences in ST knowledge scores among various groups based on wound/ostomy/incontinence specialist nurses, participation in training for wound/ostomy/incontinence care, nursing experience with patients with STs, self‐assessed grade in ST care, degree of emphasis of managers, as well as previous experience and willingness to undergo ST training.

**TABLE 4:** Comparative analysis of ST knowledge scores among nurses with different histories in ST nursing (*N* = 1293).

ST nursing histories	*N* (%)	M ± SD	*t/F*	*p*
Are you a wound/ostomy/ incontinence specialized nurse?				
Yes	304 (23.50%)	11.33 ± 2.70	13.014	<.001*
No	989 (76.50%)	8.95 ± 3.07		
Have you undergone training on wound/ stoma/incontinence nursing?				
Yes	745 (57.60%)	10.38 ± 2.91	12.022	<.001*
No	548 (42.40%)	8.34 ± 3.09		
Have you undergone specialized training on ST?				
Yes	480 (37.10%)	10.74 ± 2.88	11.318	<.001*
No	813 (62.90%)	8.78 ± 3.08		
Are you willing to participate in specialized training on ST?				
Yes	1075 (83.10%)	9.80 ± 3.05	7.541	<.001*
No	218 (16.90%)	8.07 ± 3.25		
Do you have nursing experience with patients who have ST?				
Yes	884 (68.40%)	9.86 ± 3.11	18.424	<.001*
No	342 (26.50%)	8.89 ± 2.94		
Unclear	67 (5.20%)	8.01 ± 3.78		
What is your Self‐assessed grade of ST nursing ability?				
Novice	512 (39.60%)	8.66 ± 3.33	55.988	<.001*
Advanced beginner	608 (47.00%)	9.80 ± 2.82		
Competent	104 (8.00%)	10.57 ± 2.90		
Proficient	61 (4.70%)	11.24 ± 3.09		
Expert	8 (0.60%)	14.13 ± 0.99		
Did you expecte a degree of emphasis from managers?				
Yes	1171 (90.60%)	9.72 ± 2.99	17.763	<.001*
No	51 (3.90%)	7.45 ± 3.83		
Unclear	71 (5.50%)	7.59 ± 3.99		

*Note*: *p<.05.

### 3.4 Correlation of ST knowledge status and self‐directed learning competence

**FIGURE 1:**
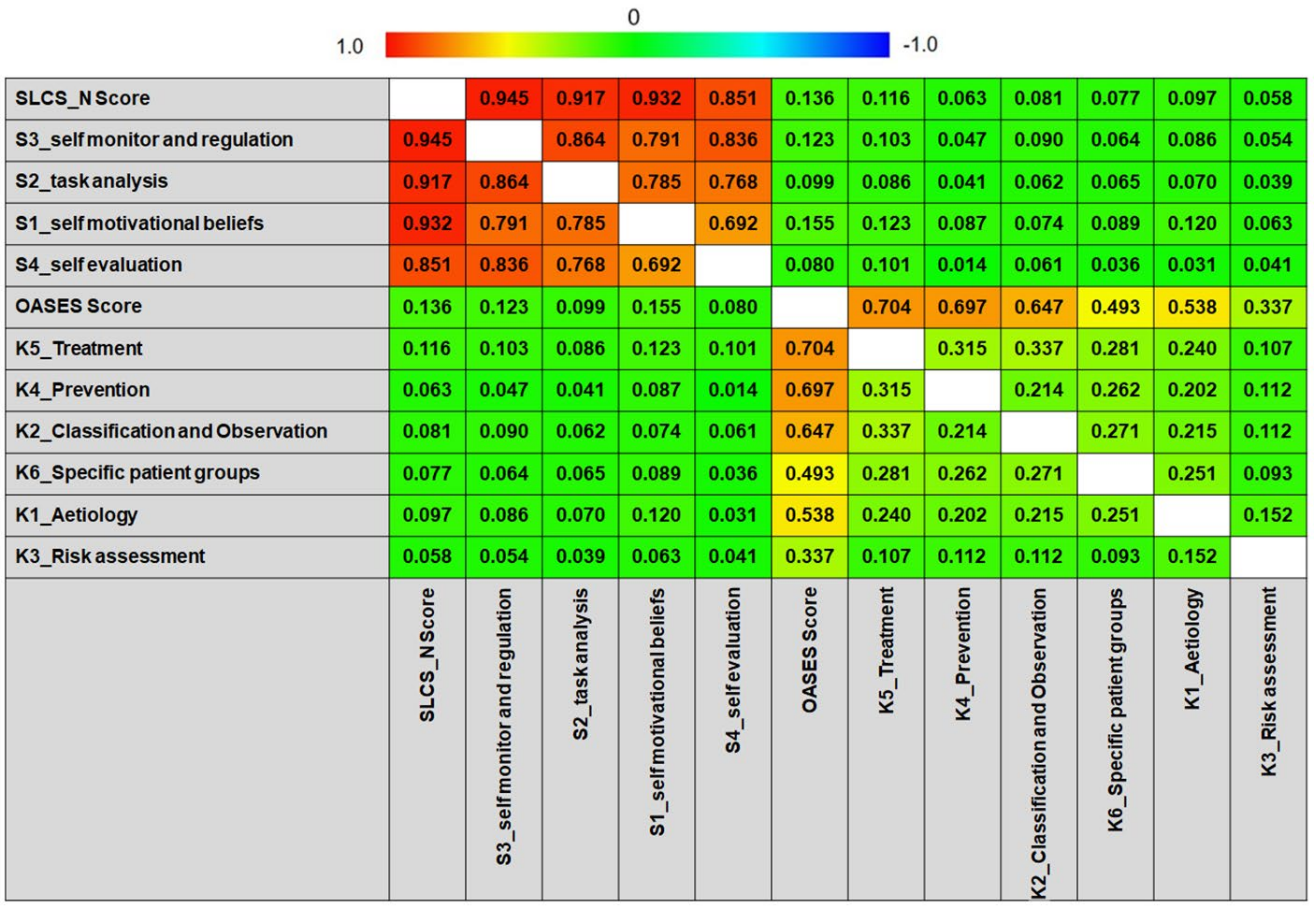
Bivariate Pearson Correlation between all dimensions of OASES and SLCS‐N scales. rom red to green to blue, *r* varies from 1.0 to 0 to −1.0. The results revealed the following: (1) a strong to extremely strong positive correlation within the dimensions of the SLCS‐N scale (*p* < .001); (2) varying degrees of correlation within the dimensions of the OASES scale, ranging from no correlation (|*r*| < .2) to a strong correlation; and (3) no correlation between the OASES and SLCS‐N scales (|*r*| < .2).

As shown in Figure 1 and Table S2, the Pearson correlation analysis revealed varying degrees of correlation within the dimensions of the OASES scale, ranging from no correlation to a strong correlation (*r* = .093–0.704, *p* <0.05), and strong to extremely strong positive correlations within the dimensions of the SLCS‐N scale (*r* = .692–.945, *p* < .001). Notably, no significant correlation was observed between the dimensions of the two scales (|*r*| < .2). After controlling for confounding effects (Table 5), the odds ratio (OR) and partial correlation analysis revealed a significant relationship between the treatment dimension in the OASES scale and all four dimensions in the SLCS‐N scale (*r* = .085–0.101, OR = 1.420–2.364, all *p* < .05), as well as between the aetiology dimension in the OASES scale and two dimensions in the SLCS‐N scale, self‐motivational beliefs, and self‐monitor and regulation (*r* = .094 and .064, OR = 3.263 and 1.840, *p* < .001 and *p* = .0023, respectively).

**TABLE 5:** The correlation degree and partial correlation coefficient of each SLCS‐N dimension with all the OASES dimensions (*N* = 1293).

Dimensions of scale	*Βeta* ± *SE*	*OR* (95%*CI*)	*r*_partial_	t	*p*
Self motivational belief					
Aetiology	1.183 ± 0.350	3.263 (1.643,6.481)	.094	3.379	<.001
Treatment	0.860 ± 0.243	2.364 (1.468,3.808)	.098	3.538	<.001
Task analysis					
Treatment	0.351 ± 0.113	1.420 (1.137,1.773)	.086	3.097	.002
Self monitor & regulation					
Aetiology	0.610 ± 0.265	1.840 (1.095,3.090)	.064	2.305	.021
Treatment	0.560 ± 0.184	1.751 (1.222,2.510)	.085	3.050	.002
Self evaluation					
Treatment	0.264 ± 0.072	1.301 (1.130,1.500)	.101	3.650	<.001

*Note*: The correlation degree was assessed using Beta and O*R* = exp (Beta). Beta>0; OR >1 is a positive correlation, and Beta<0; OR <1 is a negative correlation; *r*_partial_ is the coefficient of partial correlation, which quantifies the close extent of correlation.

### 3.5 Analysis of influencing factors of nurses' general knowledge level of ST

The independent variable assignments of demographic characteristics and ST nursing history are detailed in Table S3. Multiple linear stepwise regression analysis (Table 6) revealed six independent factors influencing nurses' overall ST knowledge scores: hospital tier, wound/ostomy/incontinence‐specialized nurses, participation in training for wound/ostomy/incontinence care, self‐assessed grade in ST care, degree of emphasis of managers and willingness to undergo ST training.

**TABLE 6:** Stepwise multiple linear regression analysis between OASES score and demographic characteristics as well as ST nursing experience (*N* = 1293).

Independent variables	*Βeta*	*SE*	*t*	*p*	*r*_partial_
Constant	−0.175	0.521	28.041	<.001*	
Hospital tier	−0.136	0.068	−5.686	<.001*	−.157
Wound/Ostomy/Incontinence specialized nurse	−0.138	0.213	−6.097	<.001*	−.168
Participation in training for Wound/Ostomy/Incontinence care	0.133	0.19	−4.632	<.001*	−.128
Self‐assessed grade in ST care	−0.144	0.101	5.036	<.001*	.139
Degree of emphasis of managers	−0.075	0.164	−5.381	<.001*	−.148
Willingness to undergo ST training	−0.175	0.225	−2.808	.005*	−.078

*Note*: *p<.05； *F* = 54.922,*R* ^2^ = .204, adjusted *R* ^2^ = .200.

## 4 DISCUSSION

This study revealed poor ST knowledge among the Chinese nurses who participated in this survey. The term ST, a specific type of skin injury, emerged in the last decade and gained significant attention in the past 5 years. The survey conducted among nurses working in hospitals at all tiers revealed that their mean overall knowledge score on ST was 9.51 ± 3.15, corresponding to a standard score rate of 47.55%. This finding is in accordance with the results obtained from emergency hospital nurses by Kacmaz et al. (Kaçmaz et al., 2023), but is lower than that of nurses working in Class III Grade A hospitals in China (Feng, Hu, & Li, 2023). Further analysis showed that the ST knowledge levels of nurses in Class III Grade hospitals in this study were consistent with those reported by Feng et al. (Feng, Hu, & Li, 2023). The ST knowledge of nurses is very poor, particularly for nurses in Class I or II hospitals.

Among the six dimensions of the OASES scale, the ‘specific patient group’ had the highest score (standard score rate of 84%). This could potentially be attributed to the accumulated experience of nurses in caring for other types of skin injuries; the more fragile skin of older and neonatal patients (Beeckman et al., 2024). Meanwhile, the dimension ‘risk assessment’, with two items of ‘long‐term use of corticosteroids’ and ‘neonatal skin physiological characteristics’, had the lowest score, indicating a very poor awareness of ST risks among nurses. Studies have been limited on ST epidemiology and risks in other cohorts (e.g. infants), except in older inpatients (Chang et al., 2016; Lopez et al., 2011; Miles et al., 2022; Serra et al., 2018; Soh et al., 2019). Additionally, the three dimensions of ‘prevention’, ‘treatment’ and ‘classification and observation’ also had lower scores, suggesting a lack of knowledge on how to identify, manage, and prevent STs. Overall, the inadequate knowledge of STs underscores a deficiency in overall training on ST care. Therefore, hospitals should prioritize structured ST education from management to enhance nurses' knowledge of this topic. Furthermore, the lack of available tools may be the primary reason for significant deficiencies in identifying ST risks among nurses. Therefore, understanding the ST risk assessment tools on the ISTAP website (https://www.skintears.org/resources) should be encouraged, and further comprehensive ST risk factors should be explored. Therefore, the integration of risk screening into routine nursing practices is recommended to enhance nurses' abilities to assess risks in clinical settings.

Generally, the acquisition and storage of nurse‐related professional knowledge systems are significantly influenced by their humanistic characteristics and professional experience. Highly skilled individuals and those with extensive vocational experience tend to prioritize the acquisition and accumulation of specialized knowledge to foster more profound comprehension within their respective fields. The present study has shown that higher hospital tiers are associated with higher total OASES scores, which is an independent factor. A systematic review confirmed that the hospital tier is a key influencing factor in nurses' knowledge of other skin injuries (Parvizi et al., 2023). Notably, the current study revealed, for the first time, that hospital tier also independently influences nurses' ST knowledge scores. Therefore, Class I and II hospitals must prioritize ST training for nurses, establish collaborative relationships with tertiary hospitals, and leverage the expertise of tertiary hospitals to bridge knowledge gaps.

The analysis also revealed independent factors influencing OASES scores from ST nursing history, including wound/ostomy/incontinence specialized nurses, participation in training for wound/ostomy/incontinence care, self‐assessed grade in ST care, degree of emphasis of managers and willingness to undergo ST training. Of these, specialized wound/ostomy/incontinence nurses and self‐assessed grades in ST care as factors influencing the OASES score have been reported previously (Dong & Meng, 2022; Lopez et al., 2011; Parvizi et al., 2023; Serra et al., 2018). Based on Benner's theory, ST nursing ability is a dynamic process influenced by the interaction of knowledge and practical experience (Benner, 1982), a specialized nurse possesses higher professional, theoretical, and practical qualities in skin management (Payne & Martin, 1993). Therefore, considering the general lack of ST knowledge among non‐specialist nurses, it would be advantageous for experienced specialist nurses to take the lead in education within hospitals.

Notably, participation in ST‐related training, which has been previously reported to be correlated with the ST knowledge score (Feng, Hu, & Li, 2023; Lopez et al., 2011; McTigue et al., 2009) and was also supported by the univariate analysis, did not retain its significance in the multivariate analysis because of the overshadowing effect of the willingness to undergo ST training. Overwhelmingly, 83.1% of the nurses expressed readiness to engage in ST training, highlighting the lack of standardized management and training systems for STs in China. Several factors contribute to this situation. First, ST is a skin injury that is easily confused with Pressure Injury (PI) (Völzer et al., 2023). Second, ST has been referred to as “the overlooked injury” and hospital nursing managers assign a low level of significance to an ST (Palmer, 2021; Scheele et al., 2020). Third, ST‐related training is scarce or absent in wound training courses (Yang, 2023); even if ST knowledge is included in wound care training, it is fragmented without comprehensiveness and scientific objectives (Kielo‐Viljamaa, 2022). This incomplete training system ultimately results in intangible outcomes, potentially accounting for ST training experience not being significant in the multivariate confounding analysis.

Regardless of any investigations into individual factors among nurses, this study is the first to propose that the primary cause of poor ST knowledge in nurses lies in management's emphasis and administration. The best practices and consensus on STs suggest that it should be given the same attention as PI and be prevented and managed scientifically (LeBlanc, 2023). Currently, STs are used as a benchmark for nursing quality in hospitals in Australia with a robust reporting and management system in place (Rayner, 2019). Edwards et al. further validated that a positive management attitude could enhance the adherence of nurses to ST prevention protocols (Edwards et al, 2017) Therefore, the most effective strategies for ST prevention in China would involve managers promoting widespread ST knowledge training at the policy and organizational levels; implementing a comprehensive ST risk assessment, prevention, reporting, and management system; and encouraging nurses to transition from having positive attitudes towards ST to evidence‐based nursing practices. This shift can ultimately enhance the quality of ST prevention and management.

Through the analysis of OASES scores across dimensions and influencing factors, it was presumed that nurses' ST knowledge primarily stems from their attention to STs. Therefore, the SLCS‐N scale was incorporated and its correlation with the OASES scale was examined. The results confirmed that the independent learning ability of nurses was not associated with their ST knowledge status. This contrasts with the results of most reports (Dong & Meng 2022; Imjai et al., 2024). In this study, a significant Pearson's correlation in the SLCS‐N scale suggests a clear causal relationship for all dimensions. There was nil to moderate correlation among the three dimensions of aetiology, risk assessment, and specific patient groups, independently, as well as between these three dimensions and the other three dimensions, which suggests that nurses may have limited access to knowledge or pay less attention to these three dimensions. Further analysis revealed that all dimensions of the SLCS‐N showed an independent correlation with the treatment and/or aetiology of OASES. These correlations support the hypothesis that the acquisition and retention of ST knowledge can be attributed to clinical practice experience. Essentially, autonomous learning of ST theory did not result in a comprehensive increase in ST knowledge but rather led to variations in ST treatment measures and understanding of aetiology. This outcome can be easily understood in the context of the lack of systematic management and training systems for STs in most domestic hospitals. Currently, most domestic nurses acquire knowledge about STs through clinical practice, focusing on tracing the aetiology and identifying treatment methods for patients with STs without paying attention to overall ST knowledge. Therefore, wound care education must include a comprehensive and professional ST curriculum system, develop a standardized ST nursing process, and provide systematic training to enhance nurses' ST knowledge, self‐directed learning competencies, and clinical nursing skills.

## 5 CONCLUSION

ST knowledge of nurses, in China, is generally deficient, particularly in primary hospitals. Multiple factors were found to independently influence nurses' ST knowledge status, including hospital tier, specialist position, other skin injury training, self‐assessed grade, training willingness and the emphasis and administration from management. Of these, emphasis and administration from management may be the primary cause.

Globally, several studies reported poor ST knowledge among nurses. Therefore, it is important to provide specialized training in ST knowledge. However, insufficient emphasis from managers may result in a lower rate of awareness and exposure to STs, thereby posing a threat to patient safety in hospitals. Future studies should focus on establishing a scientific and systematic management system for STs, particularly emphasizing its utilization as a performance indicator of hospital‐acquired adverse events and as a safety goal for nursing quality. Additionally, intensified training efforts may effectively enhance nurses' comprehension and proficiency in ST nursing practice, thereby bridging the gap between theoretical knowledge and practical application.

## AUTHOR CONTRIBUTIONS

The study was conceived and designed by FH and QJ. Data were acquired by HH, KJ, MW, and XH, while RH translated the OASES scale into Chinese. The analysis and interpretation of data as well as the writing of the main manuscript text were conducted by QJ and HH. FH and YY provided critical revisions for important intellectual content. Figure 1 was graphed by YY. Funding support was obtained by FH, QJ, and YY. All authors reviewed the manuscript.

## FUNDING INFORMATION

This research was financially supported by the Special Scientific Research Project on Wound by the Sichuan Medical Association (Taige) [Approval No: 2023TG24], the project of Sichuan Tianfu Aging Industry Development Research Center [Approval No: TFLLCY2318], Sichuan Medical Association Wound (Taige) Special Scientific Research Project [Approval No: 2021TG02], and the Basic Application Project of the Science & Technology Department of Sichuan Province [Approval No: 2019YJ0701]. The funders had no role in study design, data collection and analysis, decision to publish, or preparation of the manuscript.

## CONFLICT OF INTEREST STATEMENT

The authors declare no conflict of interest.

### PEER REVIEW

The peer review history for this article is available at https://www.webofscience.com/api/gateway/wos/peer‐review/10.1111/jan.16353.

## Supporting information


Table S1.
Table S2.
Table S3.

## Data Availability

All data generated or analysed during this study are included in this published article and its supplementary information files.
